# Reducing Virus Infection Risk in Space Environments through Nutrient Supplementation

**DOI:** 10.3390/genes13091536

**Published:** 2022-08-26

**Authors:** Hui Li, Ya-Wen Xue, Yuan Quan, Hong-Yu Zhang

**Affiliations:** Hubei Key Laboratory of Agricultural Bioinformatics, College of Informatics, Huazhong Agricultural University, Wuhan 430070, China

**Keywords:** space environment, viral infection, nutrients, methylation, mendelian randomization

## Abstract

Space exploration has brought many challenges to human physiology. In order to evaluate and reduce possible pathological reactions triggered by space environments, we conducted bioinformatics analyses on the methylation data of the Mars 520 mission and human transcriptome data in the experiment simulating gravity changes. The results suggest that gene expression levels and DNA methylation levels were changed under the conditions of isolation and gravity changes, and multiple viral infection-related pathways were found in the enrichment analysis results of changed genes including Epstein Barr virus (EBV) infection, Hepatitis B virus (HBV) infection, Herpes simplex virus (HSV) infection and Kaposi’s sarcoma-associated herpesvirus (KHSV) infection. In this study, we found that Epigallocatechin-3-gallate (EGCG) and vitamin D are helpful in reducing viral infection risk. In addition, the causal associations between nutrients and viral infections were calculated using Two sample Mendelian Randomization (2SMR) method, the results indicated that vitamin D can reduce EBV infection and HBV infection risk. In summary, our study suggests that space environments increase the risk of human viral infection, which may be reduced by supplementing EGCG and vitamin D. These results can be used to formulate medical plans for astronauts, which have practical application value for future space exploration.

## 1. Introduction

Since the end of the Apollo mission to the moon, Mars has become the target of the next phase of human space exploration. However, space exploration has brought many challenges to human physiology. For example, isolated long-term spaceflight environments have some effects on health, such as psychiatric disorders, circadian disruption, temporal dynamics of gut microbiota, immune responses, and physical-activity-related neuromuscular performance [1]. Exposure to microgravity results in loss of muscle mass, volume and performance, especially in the legs [2,3,4]. Space radiation exposure causes central nervous system damage [5], DNA damage and oxidative stress including mitochondrial damage, inflammation and chromosomal aberrations [6,7], and increases the risk of cancer [8] affecting the cardiovascular system [9]. Some studies put forward that telomere length tended to be shorter after spaceflight than before, signifying persistent DNA damage [6]. High levels of environmental noise in spacecraft can cause cardiovascular damage, sleep disturbances and cognitive deficits [10]. Due to the limited effectiveness of the air circulation system, there is an elevated CO_2_ allowance on board that also contributes to the elevation of CO_2_ in the atmosphere around the crews [11].

Immune dysfunction occurs during space flight [12], which is characterized by changes in immune cell function and reactivation of latent viruses [13]. Diseases caused by immunosuppression may occur during prolonged flight missions, including bacterial, fungal, and viral infections [14]. According to relevant statistics, in NASA’s 106 spaceflights up to 2012, 29 astronauts suffered from infectious diseases in space (i.e., eight people with a fever/chills, five people with a fungal infection, three people with a flu-like illness, four people with a urinary tract infection, three people with aphthous stomatitis, two people with viral gastroenteritis, two people with a subcutaneous skin infection, and two people with other viral diseases) [15]. Dormant viruses respond to the stress of spaceflight, such as viruses such as herpes simplex that are reactivated in humans during spaceflight [16,17,18]. Herpes virus is a highly prevalent virus that has been co-evolved with humans for thousands of years, after the primary infection, the herpes virus is in the incubation period for life and is usually asymptomatic in immune-competent individuals. In some cases, herpes virus reactivation may eventually lead to symptoms such as specific dermatitis in astronauts [19]. One study has reported that 47 of 89 (53%) astronauts from the Space Shuttle and 14 of 23 (61%) astronauts from the ISS shed one or more herpes viruses in saliva/urine samples [16]. Mehta et al., detected EBV, varicella-zoster virus (VZV) and HSV1 in the saliva of astronauts and detected Cytomegalovirus (CMV) in the urine of astronauts [17]. Stowe et al., detected gene-replicating viral transcripts of EBV in peripheral blood B lymphocytes of six astronauts on short-term space missions (about 11 days) and six astronauts on long-term missions on the ISS (about 180 days) [18].

In addition, microgravity affects the virulence of potential microbial pathogens. Bacteria appear to thrive in weightless environments—pathogens can develop thicker cell walls [20], and ongoing research suggests that enhanced bacterial virulence in space may reduce the effectiveness of antibiotic treatment [21].

Aerospace medicine is the medical assurance for astronauts exploring space, which mainly includes screening, providing medical services, and maintaining the long-term health of space travelers [22]. Considering the long distance from the earth, the power, weight and volume of the spacecraft, and the crew configuration, medical services are greatly restricted, meaning prevention through screening may be the most effective way to reduce the major physiological space flight risks [23,24]. The long-term, isolated and limited environments of space flight can adversely affect the health and performance of the crew; U.S. National Academies pointed out in the report that behavioral and mental health issues will be increasingly important during such missions [25]. The NASA twin studies have shown that space flight causes genome-wide changes in DNA methylation levels [26]. Astronauts experience a variety of stress combinations, including social isolation, lockdown, microgravity and cosmic radiation during space flight [1]. The influence mechanism of each stressor has not been determined.

In order to alleviate the above problems, this study obtained the epigenomic data (DNA methylation data) of Mars 520 mission astronauts from NASA GeneLab (https://genelab.nasa.gov/, accessed on 15 June 2021) and the immune cell expression microarray data in the gravity change environment from GEO DataSets (http://www.ncbi.nlm.nih.gov/geo, accessed on 15 June 2021) for differential analysis. Multiple viral infection pathways were found in the Kyoto Encyclopedia of Genes and Genomes (KEGG) enrichment results, indicating that multiple stressors in simulated space environments may lead to human immune disorders and increase human susceptibility to viruses. In this study, the interactive target data of nutrient elements in food from the database STITCH (http://stitch.embl.de/, accessed on 29 April 2020) and the genetic data associated with viral infections from DisGeNET (https://www.disgenet.org/, accessed on 29 April 2020) were used for performing hypergeometric tests. The results showed that Epigallocatechin-3-gallate (EGCG) and vitamin D may contribute to the resistance to the above viral infections. Many studies have reported that vitamin D has a positive effect on astronauts. Vitamin D has been used as a supplement for astronauts, and our results support previous studies. In addition, two sample Mendelian Randomization (2SMR) method was used in this study to calculate the causal association between nutrients and viral infection diseases, and the results showed that vitamin D levels were negatively correlated with levels of characteristics of Epstein Barr virus (EBV) infection and levels of characteristics of Hepatitis B virus (HBV) infection, suggesting that vitamin D may help reduce EBV infection and HBV infection.

## 2. Materials and Methods

### 2.1. Differential Methylation Analysis

DNA methylation is one of the most stable and prevalent epigenetic mechanisms and can be used to outline the underlying biological mechanisms [27,28]. The purpose of methylation differential analysis is to study the adverse effects of long-term, isolated and limited environments on the health of the crew during space flight. In this study, DNA methylation data of the Mars 520 mission (GeneLab ID: GLDS-140) were collected from NASA GeneLab (https://genelab.nasa.gov/, accessed on 15 June 2021), six astronauts experienced 520 days of high-fidelity simulated flight to Mars. Ecological validity of the simulation included a spaceship-like habitat; continuous isolation from earth’s environment; realistic mission activities; a mid-mission landing on a simulated Mars surface; accurate mission duration and timeline; operations between crew and mission controllers; communication delays inherent in interplanetary travel; limited consumable resources; exercise equipment for physical fitness; diurnal weekly work schedule; crew control of habitat lighting; and video monitoring of crew in habitat common areas [29].

Methylation differential analysis is an effective method to predict disease risk [30,31,32]. The methylation chip data of astronauts’ peripheral blood cells were downloaded at six time points during the mission, and astronaut methylation levels were calculated at six time points during the Mars 520 mission. Methylation data from six astronauts at different time points served as experimental controls. The genes with significant differences in methylation sites were calculated, and the differentially methylated genes were subjected to pathway enrichment analysis.

The differential methylation calculation is based on methylation probe annotation. The R language package “minfi” was used to read and filter probes from the original data of the methylated chip [33] and we converted the original methylation matrix to the methylation signal values. The “DMPFinder” function was used to perform differential methylation analysis on the matrix, and the “type” parameter was set as “continuous”. Significant sites (Q-value < 0.05) were screened out from the results of differential methylation analysis and differential methylation genes were obtained by site annotation.

### 2.2. Differential Expression Analysis

In this study, we collected two groups of gene expression microarray from GEO DataSets (http://www.ncbi.nlm.nih.gov/geo, accessed on 15 June 2021), which were human Jurkat T lymphocytes (GSE101102) and human U937 myelomonocytic (GSE101309).

Jurkat T gene expression microarray (GSE101102): The Jurkat T gene expression microarray is composed of the subseries of GSE94253, GSE94255 and GSE101101. During the short-term parabolic flight (GSE94253), Jurkat T cells underwent a 20 s high-gravity lift-off and immediately followed by a 20 s microgravity parabolic flight; during the rocket launch (GSE101101), Jurkat T cells underwent a 75 s high-gravity and immediately followed by a 340 s microgravity.

U937 gene expression microarray (GSE101309): The U937 gene expression microarray is composed of the subseries of GSE101212 and GSE101299. Same conditions as Jurkat T, during the short-term parabolic flight (GSE101212), U937 myelomonocytic underwent a 20 s high-gravity lift-off and immediately followed by a 20 s microgravity parabolic flight; during the rocket launch (GSE101299), U937 myelomonocytic underwent a 75 s high-gravity and immediately followed by a 340 s microgravity.

We also conducted transcriptome analysis on T cells (GSE38836) from non-cancer patients, the T cells from non-cancer patients (GSE38836) were sent into space. This part of the analysis would be useful to compare the transcriptional levels of T cells in non-cancer and cancer patients.

We conducted a differential analysis on the gene expression microarray mentioned above. The normalized gene expression data was downloaded and extracted using the R language “GEOQuery”. The “limma” packet of R language was used for differential analysis and significant loci were selected (adjust *p*-value < 0.05). The “clusterprofiler” program package of R language was used to perform functional enrichment analysis on differentially expressed genes to determine which metabolic pathways these different genes were located in.

### 2.3. Viral Infection-Related Nutrient Enrichment Analysis

The nutrient enrichment analysis was based on the conclusion of nutrigenomics [34], and the hypergeometric test method was used to conduct enrichment analysis of the nutrient-related gene set and the disease-related gene set [28]. The idea behind this approach is that food compounds and nutrients may exert their potential health benefits by acting on genes associated with disease [35,36]. In this study, we collected 235 nutrients in food from the United States Department of Agriculture (USDA) (https://www.usda.gov/topics/organic, accessed on 29 April 2020). Proteins with interaction scores greater than 0.400 interacting with nutrients were extracted from the STITCH database (http://stitch.embl.de/, accessed on 29 April 2020), and 46,907 interactive relationships between 130 nutrients and 8408 protein targets were finally obtained. Thirty-five viral infectious diseases and their gene-disease associations (GDAs) were extracted from DisGeNET (https://www.disgenet.org/, accessed on 29 April 2020). Subsequently, using the “phyper” function of the stats program package of R language to perform a hypergeometric distribution test to calculate which gene sets of 130 nutrients were significantly enriched in the GDAs of the 35 viral infection diseases, and the “p.adjust” function was used to perform FDR correction for the *p*-values of the results and significant loci were selected (adjust *p*-value < 0.05). If a nutrient-related gene is significantly enriched in the viral infection pathway, there is a high probability that this nutrient interacts with the genes in the viral infection pathway [34,37].

### 2.4. Two Sample Mendelian Randomization Analysis

Mendelian randomization (MR) uses known SNP–phenotypic relationships and SNP–disease relationships to examine possible phenotypic disease causality. Typically, 2SMR uses independent Genome Wide Association Study (GWAS) data from phenotypes and diseases to test their causal effect with a single SNP as an instrumental variable (IV). In this study, we study the causal relationship between nutrients and diseases based on the 2SMR method.

The 2SMR method first requires the preparation of exposure factors and outcome variables. According to the results of methylation analysis and transcriptome analysis, the viral pathways significantly enriched included EBV, HBV, Herpes simplex virus (HSV) and Kaposi sarcoma-associated herpesvirus (KHSV). Summary data for GWAS related to the above viral infectious diseases were collected as outcome variables from the IEU GWAS database (https://gwas.mrcieu.ac.uk/, accessed on 8 October 2020). Here we obtained three results related to EBV in total: ebi-a-GCST006362 (GWAS ID: ebi-a-GCST006362), Anti-Epstein-Barr virus nuclear antigen (EBNA) IgG levels (GWAS ID: ebi-a-GCST006362), Anti-Epstein-Barr virus early antigen (EA) IgG levels (GWAS ID: ebi-a-GCST006345); three results related to HBV in total: Chronic hepatitis B (GWAS ID: bbj-a-99), Anti-hepatitis B virus surface antigen (HBs) IgG levels (GWAS ID: ebi-a-GCST006354), Anti-hepatitis B virus core antigen (HBc) IgG levels (GWAS ID: ebi-a-GCST006355); and two results related to Human papillomavirus (HPV): HPV E7 Type18 (GWAS ID: prot-c-2624_31_2), HPV E7 Type 16 (GWAS ID: prot-c-2623_54_4). There is no HSV and KHSV data in the database.

With nutrients as exposure, a total of 13 SNPs significantly associated with nutrients were collected in the GWAS Catalog (https://www.ebi.ac.uk/gwas/, accessed on 8 October 2020). SNPs need to be strongly associated with nutrients as exposure factors. Therefore, only the SNPs with very significant correlations (*p*-value < 5 × 10^−8^) were retained, the SNPs with linkage disequilibrium (LD) effect were deleted, and SNPs significantly associated with other confounding factors were filtered out using R language “phenoscanner” package. After the above processing, we obtained four SNP data sets significantly related to nutrients from GWAS Catalog, they are vitamin D Levels (GCST002602), calcium levels (GCST002201), selenium levels (GCST002671) and ferritin levels (GCST002677).

Additionally, we used the “harmonise_data” function of the “TwoSampleMR” package to harmonize these SNPs and ensure that the SNPs’ effect on exposure and outcome corresponds to the same allele, respectively. Finally, the “mr” function was used to conduct a Mendelian randomization analysis on the processed data. In this part, Inverse Variance Weighting (IVW), Weighted Median (WM) and MR Egger regression were used as the main causal effect estimation. Among them, the IVW method focuses on integrating multiple genetic variants to infer the causal relationship between exposure factors and outcome variables. It is a weighted linear regression of Walder ratios calculated from different SNPs to obtain the causal relationship of unbiased estimation. WM is a weighted density function for ratio estimation. Multiple genetic effect values can be integrated by assigning different weight values to each genetic variable. If at least half of the information in the analysis comes from valid instrumental variables, then the causal effects can be continuously estimated. MR Egger takes gene pleiotropy into account, and the principle is the same as for IVW, but the difference is that the intercept term for IVW must be 0. In contrast, it is not necessarily the case for MR Egger, where the intercept is used to check whether there is a genetic pleiotropy. When the intercept corresponds to a statistic with a *p*-value > 0.05, it means that the causal effect is not influenced by genetic pleiotropy.

## 3. Results

### 3.1. Differential Methylation Analysis of Mars 520 Samples

**Figure 1:**
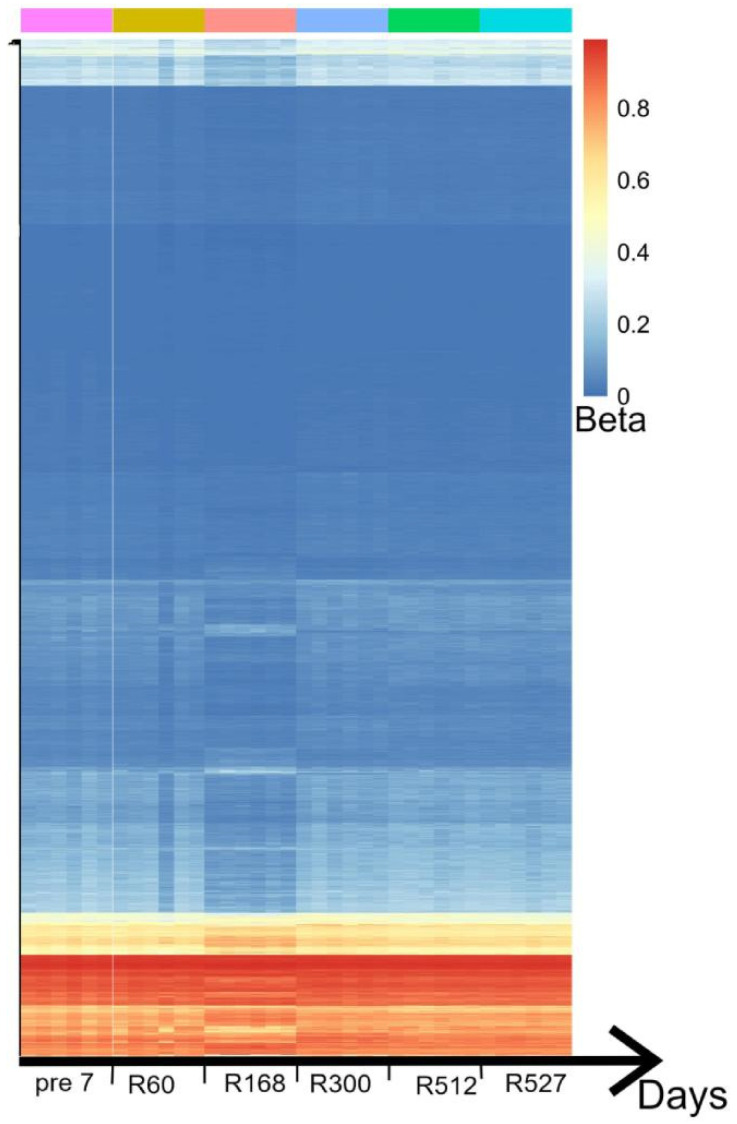
Heatmap of differential methylation probes in Mars 520 samples. The differential methylation analysis of Mars 520 samples resulted in 6470 DMPs. The horizontal axis shows the time change, six time points in the Mars 520 sample: Pre7: One week before the mission; R60: Day 60 of the mission; R168: Day 168 of the mission; R300: Day 300 of the mission; R512: Day 512 of the mission; R527: Day 527 of the mission. Each column is a sample, and each row is a DMP. The β values of DMPs represent the levels of methylation of probes. On day 168, the methylation levels of the gene changed significantly.

DNA methylation is a relatively stable epigenetic mechanism [27], under long-term isolation and strict condition controls, the methylation differential analysis results showed that the methylation levels of the six astronauts changed significantly with time. On the 60th day after the Mars 520 mission, the methylation levels of genes in the astronauts’ peripheral blood cells began to change slightly; the methylation levels of genes changed significantly on the 168th day. As the mission progressed, the methylation levels of most genes stabilized, possibly reflecting the astronauts’ gradual adaptation to the mission environment. However, there are still a few genes in the abnormal methylation state, which indicates that long-term isolation may have a lasting effect on some genes (Figure 1).

**Figure 2:**
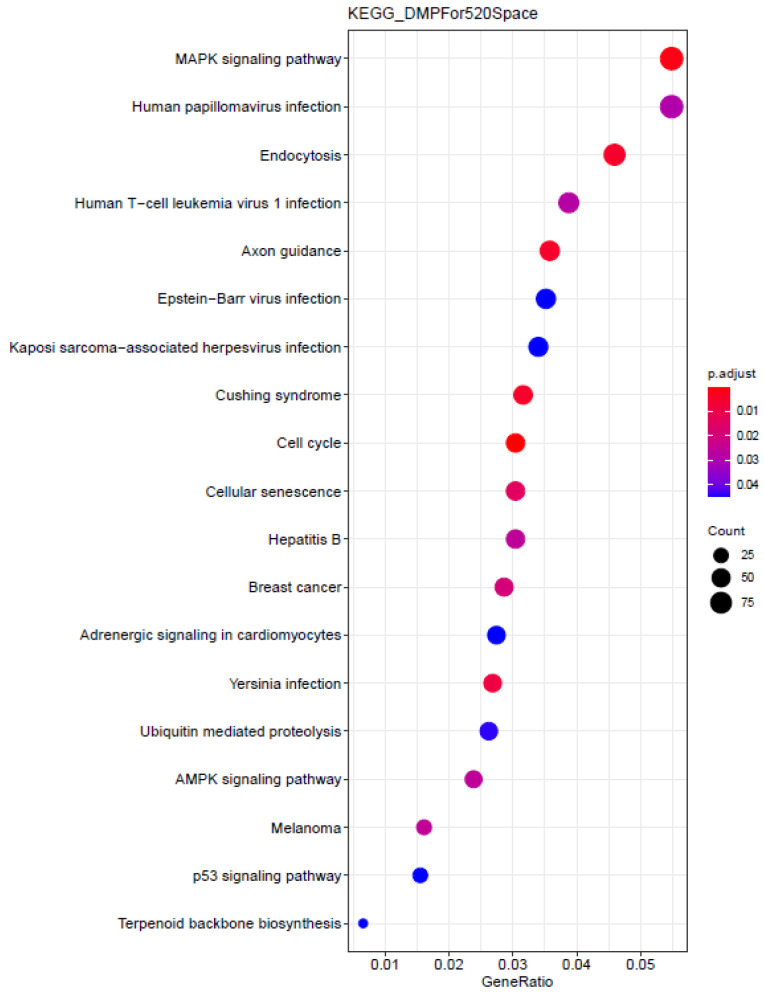
KEGG enrichment pathways of differential methylated genes in Mars 520. The KEGG enrichment analysis of 4428 differentially methylated genes included 5 pathways associated with viral infections, including Human papillomavirus (HPV) infection, Human T cell leukemia virus 1 (HTLV1) infection, Epstein Barr virus (EBV) infection, Kaposi Sarcoma-Associated Herpesvirus (KSHV) infection and hepatitis B. The horizontal axis represents GeneRatio, which is the ratio of genes enriched in the corresponding pathway to the input genes; the dot size in the figure was determined by the number of genes enriched in the corresponding pathway and the dots in the figure were arranged from top to bottom as number became smaller; the color is determined by “adjust *p*-value”, which was the false discovery rate provided by the KEGG enrichment tool.

In this study, 6470 differential methylation probes (DMPs) were obtained by differential analysis on the methylation data of Mars 520 samples (Q-value < 0.05). Subsequently, 4428 differential methylation genes were obtained by annotating these 6470 DMPs and subjected to GO and KEGG enrichment analysis. The results were shown in Figure 2. In the KEGG significant enrichment results (Figure 2), up to five viral infection pathways and one bacterial infection pathway were found: HPV infection, EBV infection, HBV infection, Human T cell leukemia virus 1 (HTLV1) infection, Kaposi Sarcoma-Associated Herpesvirus (KSHV) infection and Yersinia infection, respectively, indicating that astronauts on such long-term space flights may be at higher risk of viral infection.

### 3.2. Differential Gene Expression Analysis of Immune Cells

In this study, the differential expression analysis was performed on human Jurkat T lymphocytes (GSE101102) and human U937 myelomonocytic (GSE101309) under simulated gravity change.

**Figure 3:**
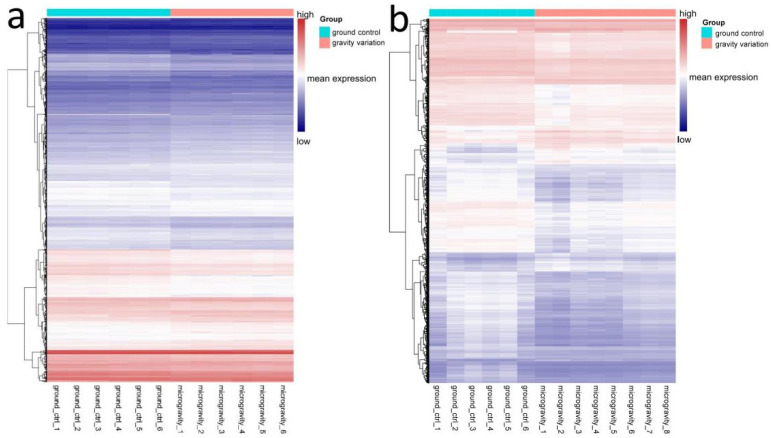
Heatmap for gene expression microarray of U937 cells and Jurkat T cells. Differential expression analysis was performed on Jurkat T cells (**a**) and U937 cells (**b**) samples, which were divided into gravity change group and ground control group. In the gravity variation group, U937 cells and Jurkat T cells underwent a 40 s gravity change flight, including a 20 s high-gravity lift-off and immediately followed by a 20 s microgravity parabolic flight. 6095 and 2970 differentially expressed genes were obtained in U937 and Jurkat T, respectively. Genes in the two groups of cells were expressed differently in microgravity conditions. Each line of the heat map represents a gene. The text below is the sample number, each column of the heat map represents a sample. The left tree structure represents a similar settlement relationship between samples. In the picture, white represents the mean expression of the sample, red represents the genes with high expression levels and blue represents the genes with low expression levels.

In human Jurkat T lymphocytes: during the short-term aircraft flight, Jurkat lymphocytes underwent a 40 s gravity change flight, including a 20 s high-gravity lift-off and immediately followed by a 20 s microgravity parabolic flight. There are 13095 significantly differentially expressed probes (adjust *p*-value < 0.05) and 6095 differentially expressed genes were obtained through annotation (Figure 3a). Jurkat T lymphocytes underwent a 75 s high gravity and immediately followed by a 340 s microgravity in another set of experiments and only 1 probe with a significant difference in methylation was found compared to the ground control group.

In human U937 myelomonocytic: during the short-term aircraft flight, U937 myelomonocytic cells underwent a 40 s gravity change flight, including a 20 s high-gravity lift-off and immediately followed by a 20 s microgravity parabolic flight. There are 4976 significantly differentially expressed probes (adjust *p*-value < 0.05) and 2970 differentially expressed genes were obtained through annotation (Figure 3b). In another experiment, U937 myelomonocytic were subjected to 75 s high-gravity and 340 s microgravity, and no probes with a significant difference in methylation were found compared to the ground control group.

As can be seen in Figure 3, gene expression of human Jurkat T lymphocyte and human U937 myelomonocytic changed significantly under gravity change conditions.

**Figure 4:**
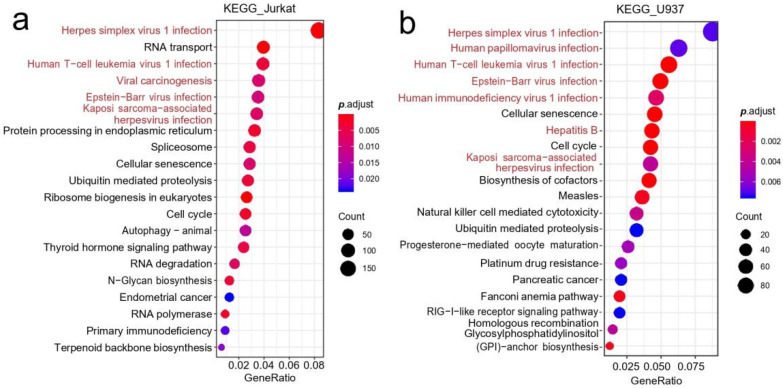
KEGG significant enrichment pathways of differently expressed genes of U937 cells and Jurkat T cells. In Jurkat T cells (**a**), five significant enrichment pathways associated with viral infection were found: HSV1 infection, HTLV1 infection, EBV infection, KSHV infection and viral carcinogenesis. In U937 cells (**b**), seven significant enrichment pathways associated with viral infection were also found: HSV1 infection, HPV infection, HTLV1 infection, EBV infection, HIV1 infection, and Hepatitis B virus (HBV) infection and KSHV infection. The horizontal axis represents GeneRatio, which is the ratio of genes enriched in the corresponding pathway to the input genes; the pot size in the figure is determined by the number of genes enriched in the corresponding pathway and the pots in the figure are arranged from the top to bottom as the number gets smaller; the node color is determined by adjusting *p*-value, which is the false discovery rate provided by the KEGG enrichment tool.

GO and KEGG enrichment analyses were performed on the above differentially expressed genes in Jurkat T and U937 cell lines, and the results were shown in Figure 4. A total of five significant enrichment pathways associated with viral infection were found in the KEGG results of differentially expressed genes in Jurkat T cells: Herpes simplex virus 1 (HSV1) infection, HTLV1 infection, EBV infection, KSHV infection and viral carcinogenesis (Figure 4a). At the same time, seven significant enrichment pathways associated with viral infection were also found in the KEGG results of differentially expressed genes in U937 cells: HSV1 infection, HPV infection, HTLV1 infection, EBV infection, human immunodeficiency virus I Type (HIV1) infection, HBV infection and KSHV infection (Figure 4b).

**Figure 5:**
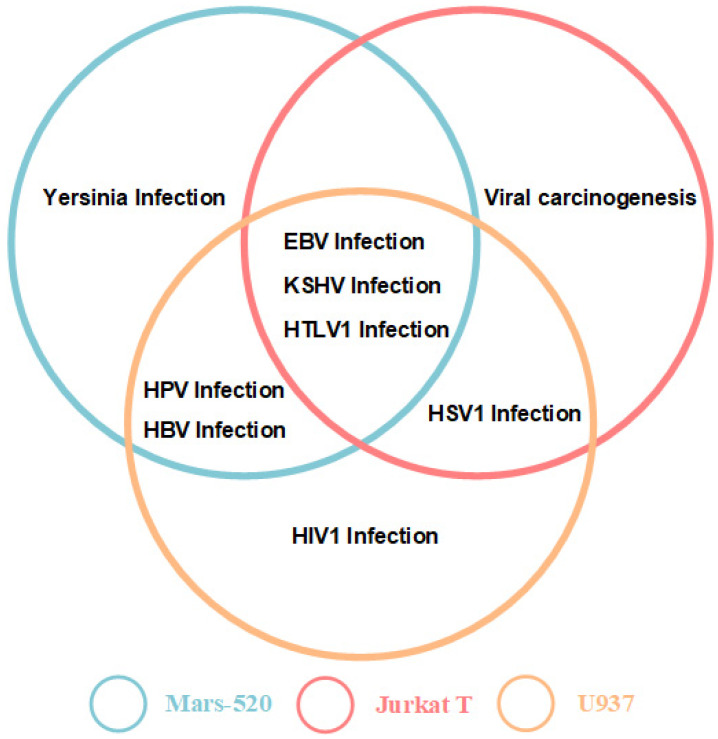
The intersection of the viral infection pathways enriched in Mars 520, Jurkat T and U937 cells. In the KEGG enrichment results of Mars 520, Jurkat T and U937 cells of differential expression genes, EBV infection pathway, KSHV infection pathway and HTLV1 infection pathway appeared simultaneously in the three groups of chips; HPV pathway infection and HBV infection pathway appeared simultaneously in Mars 520 and Jurkat T cells; HSV1 infection pathway appears in Jurkat T cells and U937 cells.

**Figure 6:**
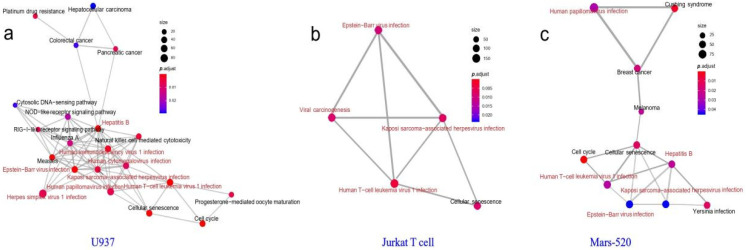
The clustering results of significantly enriched KEGG pathways. The KEGG enrichment results of data of human U937 cells (**a**), human Jurkat T cells (**b**) and Mars 520 (**c**), were clustered according to the overlap degree of differential genes. The node size in the figure is determined by the number of genes enriched in the corresponding pathway; the color changes in the figure are determined by adjusting the *p*-value, which is the false discovery rate provided by the KEGG enrichment tool; lines in the network represent there were overlapping genes in the two connected pathways, and thicker lines represent more overlapping genes in the two pathways.

It was found that the three sets of differential genes of U937 cells and Jurkat T cells, Mars 520 contain seven viral infection pathways and one bacteria pathway (Figure 5) and many crossovers (Figure 6). This may indicate that astronauts are facing a higher risk of viral infection in simulated space environments. In particular, the EBV infection pathway, the HTLV1 infection pathway and the KSHV infection pathway simultaneously appeared in the three KEGG enrichment results (Figure 5). During the transcriptome analysis, we also conducted transcriptome analysis on T cells (GSE38836) from non-cancer patients and found HBV infection pathway and HTLV1 infection pathway in the enrichment pathway. The results indicated that the space environments had similar effects on T cell lines in cancer and non-cancer patients, especially their effects on HBV infection.

### 3.3. Nutrient Enrichment Analysis

It can be inferred from the above analysis that multiple stressors in the simulated space environments may cause damage to the human immune system, exposing astronauts to the risk of viral infection and reactivation of latent viruses in the body. Some micronutrients and dietary ingredients play a very significant role in developing and maintaining the immune system and reducing chronic inflammation. For example, micronutrients vitamin A and zinc regulate cell division and are therefore necessary for a successful proliferation response in the immune system. Nutrients can also play a variety of immune roles, such as vitamin E, which acts as an antioxidant, inhibits the activity of protein kinase C and may interact with enzymes and transporters. Therefore, by identifying which nutrients help immune cells resist viral infections, these nutrients can be supplemented in a targeted manner for astronauts during space flight, thereby reducing the risk of viral infections.

**Figure 7:**
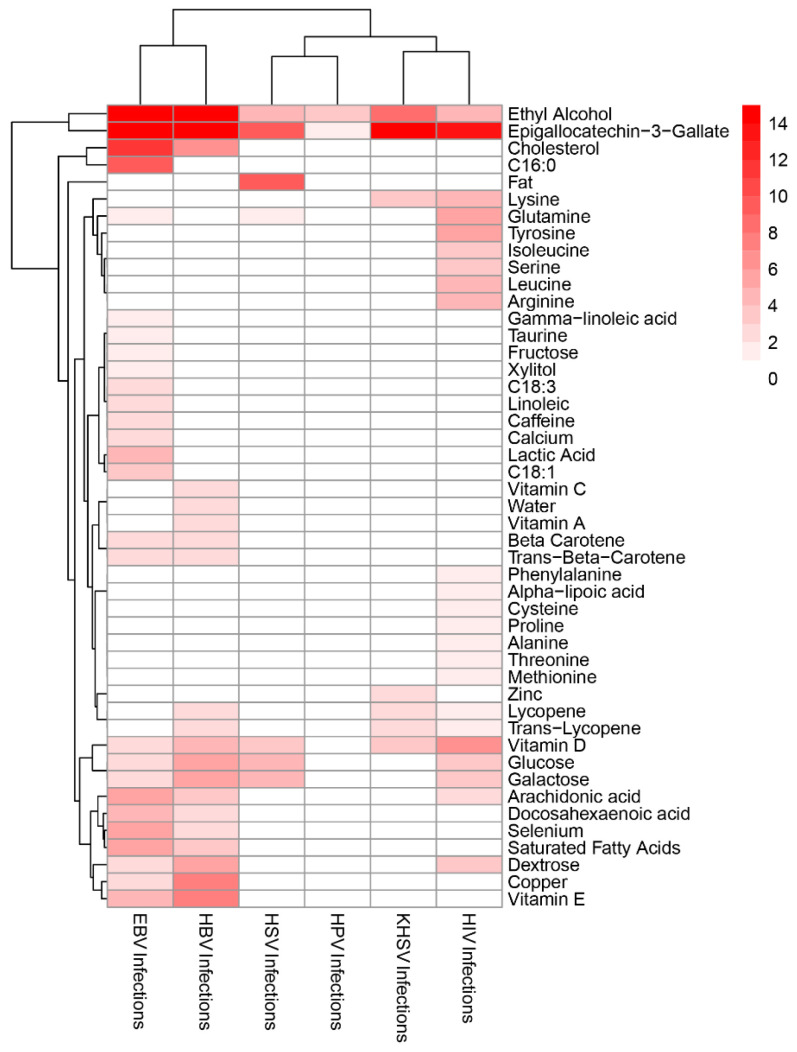
The analysis results of interaction between viral infection and nutrients. There are 47 interactions between nutrients and viral infections. The darker the color, the higher the confidence of the interactions (based on adjusted *p*-values of hypergeometric distribution tests). The scale in the figure indicates the decimal units of *p*, with the norm of adjusted *p*-value ranging from less than 1 × 10^−14^ to 1.

In this study, the hypergeometric distribution test was performed on the target gene sets of 130 nutrients in the food collected from USDA and the gene sets of 35 viral infections. If a target gene set of the nutrient is significantly enriched in a viral infection pathway, then there is a high probability that the nutrient interacts with the genes in the viral infection pathway. In the results of the hypergeometric distribution test, six viral infection pathways that were significantly enriched in the simulated spatial environmental omics data were found: HSV infection, EBV infection, KSHV infection, HIV infection, HPV infection and HBV infection, and there was a significant interaction relationship obtained between them and 47 nutrients (Figure 7). Several micronutrients, particularly EGCG, ethanol, and vitamin D, interacted significantly with multiple viral infections simultaneously (Table 1).

**Figure 8:**
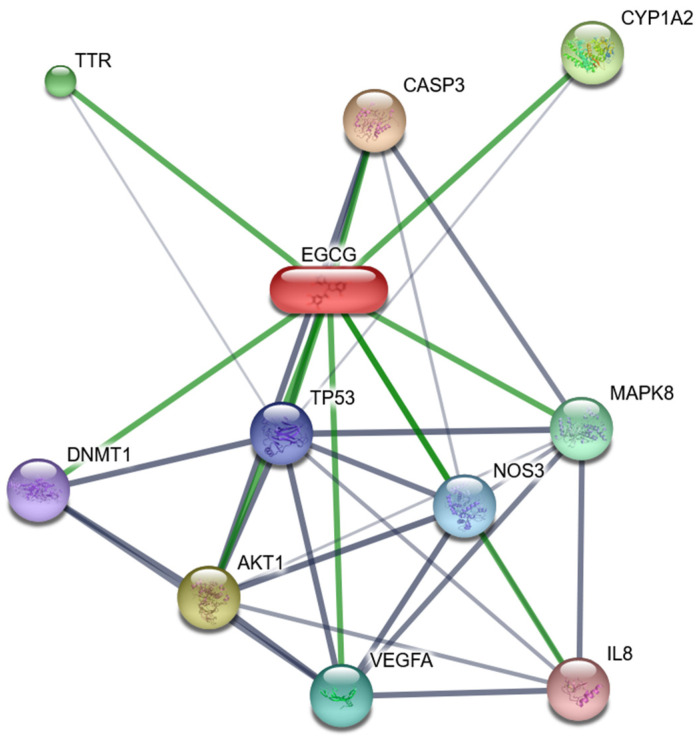
The interactive network of proteins and Epigallocatechin-3-gallate (EGCG). The interactive network of proteins and EGCG, the Interactive nodes with the top ten interaction scores were shown. Lines represent protein-protein associations that are meant to be specific and meaningful. Proteins jointly contribute to a shared function and stronger associations are represented by thicker lines. Protein–protein interactions are shown in grey, EGCG-protein interactions in green.

The natural product EGCG is an ester formed from gallic catechin and gallic acid and is the main catechin found in green tea. In recent years, many studies have reported that EGCG has anti-infective effects. EGCG interacted significantly with multiple viral infections and shows that EGCG plays a positive role in treating diseases. We obtained network nodes of the top ten scores in the EGCG interactive network (Figure 8), EGCG regulates key genes or gene expression products as follows: KEGG resulted in increased expression or activity of CASP3, TP53, MAPK8 proteins, and decreased expression or activity of AKT1, CXCL8, DNMT1 proteins. In HBV, EBV, HIV and other viral infections, viruses prevent apoptosis by enhancing the expression level of anti-apoptotic genes [38]. In addition, we also performed KEGG enrichment analysis on the EGCG-protein interactive network, and the results showed very rich KEGG enrichment results related to cancer and infectious diseases. These include Hepatitis B (false discovery rate: 8.68 × 10^−7^), Hepatitis C (false discovery rate: 2.68 × 10^−5^), pathways in cancer (false discovery rate: 8.68 × 10^−7^) and EBV (false discovery rate: 0.00139). The regulation of EGCG on related pathways can effectively fight against viral infection. For example, EGCG has a positive effect on AKT1-TP53-induced programmed apoptosis: EGCG results in decreased phosphorylation of AKT1 protein [39] and activates the expression of TP53 gene product p53. AKT1 regulates many processes including metabolism, proliferation, cell survival, growth and angiogenesis [40,41]. Dephosphorylation of ATK may be an important mechanistic step in drug-induced programmed cell death [42], reduced AKT phosphorylation can induce apoptosis of cancer cells [43] and is involved in the inhibition of tumor growth and metastasis [44]. In an experiment on radiation-induced apoptosis of salivary acinus in mice, apoptosis is reduced in transgenic mice expressing a constitutively activated mutant of AKT1 [39]. Inhibiting the expression of AKT1 and promoting the accumulation of p53 is conducive to the normal operation of apoptosis-related signaling pathways [45], which can be achieved by supplementing EGCG.

Vitamin D plays an important role in mediating the immune system’s response to infection [46]. It helps reduce the risk of microbial infection and death, mainly involving three types of functions: physical barrier, cellular innate immunity and adaptive immunity [47]. Vitamin D enhances the innate immunity of cells by inducing antimicrobial peptides while maintaining tight junctions, gap junctions and adhesion junctions [48,49]. Antimicrobial peptides have direct antibacterial effects on a variety of microorganisms, including Gram-positive bacteria and Gram-negative bacteria, enveloped viruses, and fungi [50]. The antimicrobial peptides can also induce a variety of pro-inflammatory cytokines, stimulate the chemotaxis of neutrophils, monocytes, macrophages and T lymphocytes to the infected site, and induce the apoptosis and autophagy of infected epithelial cells to help clear respiratory pathogens [51,52]. In addition, preclinical trials reported by Mansueto et al., showed that treatment of peripheral blood monocytes with 1,25 (OH) 2D (the active form of vitamin D) reduced the sensitivity of cells to HIV infection by inhibiting viral entry, regulating the expression of CD4+ cell surface antigens, inhibiting the production of viral p24, and restricting the proliferation of monocytes [53]. Studies have shown that vitamin D supplementation may help improve the clinical prognosis of patients with COVID-19 [54,55]. Lycopene has not been reported to have any antiviral activity, which may require further studies. In summary, the enrichment analysis results show that EGCG and vitamin D may enhance the body’s immunity and have the potential to reduce or resist viral infections.

### 3.4. Mendelian Randomization Causal Association Analysis

**Figure 9:**
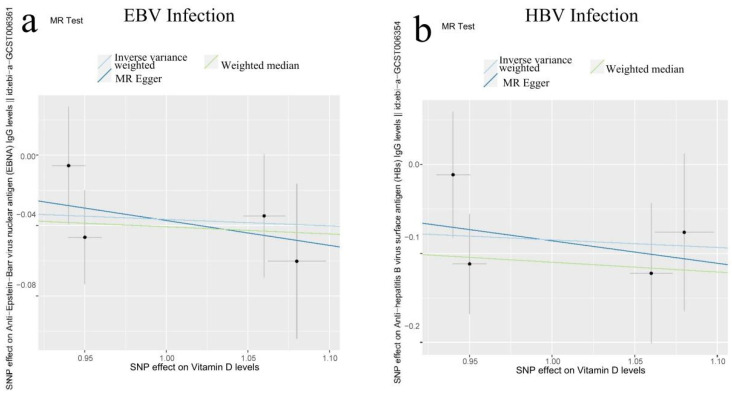
Results of Two sample Mendelian Randomization (2SMR) between vitamin D levels and indicator levels of viral infections. A causal relationship between vitamin D levels and indicator levels of EBV infection (**a**) and indicator levels of HBV infection (**b**) was calculated using the 2SMR method. We used three methods provided by the R package “TwoSampleMR”, MR Egger, Inverse variance Weighted (IVW) and Weighted Median (WM) to calculate the causal relationship between various nutrients and disease. (**a**) Results calculated by the three methods showed a negative correlation between vitamin D and indicator levels of EBV infection, suggesting that vitamin D supplementation may reduce the risk of EBV infection. (**b**) Results calculated by the three methods showed a negative correlation between vitamin D and indicator levels of HBV infection, suggesting that vitamin D supplementation may reduce the risk of HBV infection.

Mendelian randomization, as a natural experiment, uses genetic variation to investigate the causal associations between potential changeable risk factors and diseases in observational data. In this study, the 2SMR method was used to calculate the relationship between nutrients and characteristics of viral infection with SNP as a genetic tool variable, the causal relationship between three nutrients and three indicators of viral infection (EBV: EBNA IgG levels, HBV: HBs IgG levels, HPV: HPV E7 Type18 levels) was obtained. We used three methods provided by the R package “TwoSampleMR”, MR Egger, Inverse variance Weighted (IVW) and weighted median to calculate the causal relationship between various nutrients and indicator levels of viral infections. The results showed that vitamin D levels are causally and negatively correlated with EBNA IgG levels and HBs IgG levels (Table 2 and Figure 9), so vitamin D supplementation can improve EBV and HBV diseases.

EBNA IgG levels are one of the indicators for clinical detection of nasopharyngeal carcinoma, and it has been shown that high-dose vitamin D3 supplementation can reduce EBNA-1 IgG levels in patients with multiple sclerosis [56]. Clinical studies have shown that vitamin D levels are negatively related to the amount of HBV-DNA replication and are related to the prognosis of patients with liver cirrhosis [57]. Vitamin D may help reduce the risk of EBV and HBV infection, and interestingly, similar results were obtained in this study (Table 1, Figure 9).

## 4. Discussion

### 4.1. Higher Risk of Viral Infection in the Space Environments

Viruses are an essential part of the human microbiome, which can harbor viruses even during space missions. Diseases caused by immunosuppression may occur during prolonged flight missions, including bacterial, fungal and viral infections [14]. The sensitivity of the cells of the human immune system to reduced gravity has been confirmed by numerous studies in real and simulated microgravity, human immune cells are an ideal model for studying the regulation of gravity [58]. In a simulation experiment studying transcriptome changes in human myelomonocytic cell line U937 in microgravity, alterations in the transcriptome after 20 s of microgravity, more than 98% of all initially altered transcripts adapted rapidly to the new gravitational environment after 300 s of microgravity [59]. The results of our study showed that the gene expression changes in T cells and U937 cell lines showed regularity. Both Jurkat T and U937 cells showed significant changes in gene expression levels after 40 s of gravity change, while gene expression levels almost completely returned to their initial state after 5 min of gravity change. We obtained the gravity-sensitive gene set and conducted a KEGG enrichment analysis on the gene set. These gene sets were significantly enriched in the viral infection pathway, and although gene expression levels in cell lines recovered rapidly, our results indicate that astronauts are at increased risk of infection during gravity changes. Moreover, during the 520 days of simulated flight in isolation, the DNA methylation data of astronauts changed significantly, and many viral infection pathways were found in the KEGG pathway enrichment results. From our understanding, the limited isolation environment and workload may be the main reasons for the changes in the astronauts’ methylation levels. Over time, the astronauts developed sleep disturbances during the experiment [60], which may be caused by psychological factors and reduced exercise. The methylation levels of genes changed more significantly on day 168 compared with other periods, the emergence of significant changes in gene expression so late in the experimental period is in line with expectations, as a result of psychological problems caused by prolonged isolation and the accumulation of physiological load, compared to the early stage of the experiment the late changes are more pronounced. Therefore, we believe that long-term adverse stress factors and short-term gravity changes during flight will have adverse effects on the human body and increase the risk of viral infection. We also found multiple viral infection pathways in KEGG results of differentially expressed and differentially methylated genes (Figure 5) and the viral infection pathways in three sets of differential genes are highly concentrated (Figure 6), the EBV infection pathway, the HTLV1 infection pathway and the KSHV infection pathway simultaneously appeared in KEGG enrichment results (Figure 5), where EBV and KSHV were human herpesviruses and HTLV1 was a retrovirus.

In short-term and long-term spaceflight radiation and microgravity will form a unique set of stressors that can lead to dysregulation of the astronaut’s immune system and endocrine system [60,61]. When the human body is infected with a virus, suppressing the virus during the incubation period requires a strong and vigilant immune system, which is highly dependent on cytotoxic T cells. Immune disorders promote virus reactivation, which has been confirmed in terrestrial space simulation studies [62] and space studies [17,18]. In KEGG clustering results, viral infection pathways were all highly aggregated, indicating that the differential genes of these pathways were highly overlapping. In other words, abnormal gene methylation/expression induced by simulated space environments causes damage to the human immune system and may increase the risk of viral infection and reactivation of latent viruses in the body.

### 4.2. Positive Role of Vitamin D and EGCG in Reducing Viral Infection Risk

Some micronutrients and dietary ingredients play a very significant role in developing and maintaining the immune system and reducing chronic inflammation. By identifying which nutrients help immune cells resist viral infections, these nutrients can be supplemented in a targeted manner for astronauts during space flight, thereby reducing the risk of viral infections. In the analysis of viral infection-related nutrient enrichment, we found significant interaction between several micronutrients and viral infection (Figure 8), in particular, epigallocatechin-3-gallate (EGCG) interacts significantly with HSV, EBV, KSHV, HIV and HBV infection; HSV, EBV and KSHV are sporoviruses, HIV and HBV are retroviruses. Vitamin D interacted significantly with HIV, HBV, and EBV (Table 1). In addition, KEGG enrichment analysis of genes in the EGCG- gene interactive network revealed several viral infection pathways including the HBV pathway, Hepatitis C pathway, pathways in cancer and EBV pathway.

The 2SMR method was used to calculate the causal relationship between various nutrients and indicators of viral infections using SNP as a genetic tool variable. The results showed that vitamin D supplementation could reduce the infection characteristics of EBV (EBNA IgG levels) and HBV (HBs IgG levels) (Table 2 and Figure 9). It suggests that vitamin D supplementation may reduce EBV and HBV infection risk. EBNA IgG levels are one of the indicators for clinical detection of nasopharyngeal carcinoma and it has been shown that high-dose vitamin D3 supplementation can reduce EBNA-1 IgG levels in patients with multiple sclerosis [47]. Clinical studies have shown that vitamin D levels are negatively related to the amount of HBV-DNA replication and are related to the prognosis of patients with liver cirrhosis [48], similar results were obtained in this study (Table 1, Figure 9).

## 5. Conclusions

The medical services provided on the spacecraft are severely restricted. An effective way to mitigate the physical risks of spaceflight is to screen for potential diseases that may threaten the crew and take preventive measures. Especially on missions to Mars or long flights, ensuring an understanding of how the nutritional needs of astronauts differ from those on Earth and how nutrients can be used as a response to the sustained effects of the space environment on the body is an important part of space health research.

The biological data related to spatial studies are limited and the sample size is small. How to extract as much information as possible from the limited data, fully assess and reduce the physiological risks that may be caused by the space environments and improve the ability of risk prevention and control are the main challenges facing current space exploration. In order to solve this problem, the methylation data of peripheral blood cells of six astronauts in the Mars 520 project, and the gene expression data of human Jurkat T lymphocyte and human U937 myelomonocytic under microgravity collected from the NASA GeneLab database were analyzed for differential methylation and differential expression. The results showed that the differential genes in the three microarrays contained multiple viral infection pathways, such as EBV infection, HBV infection, HSV infection, KSHV infection, etc. Latent virus reactivation has also been reported in astronauts on past missions, suggesting that astronauts may be at serious risk of viral infection or reactivation during space flight. In order to find effective measures to reduce viral infections risk, this research collects nutrients in food from USDA, obtains interaction information between nutrients and protein targets, genes, and viral infections from the STITCH database and DisGeNET database, and uses hypergeometric testing to find Nutrients that are significantly related to viral infections. The 2SMR method is then used to study the causal relationship between nutrients and viral infections.

Finally, according to the results of bioinformatics analysis, vitamin D may have the effect of reducing HIV, HBV and EBV infection risk. Vitamin D has been used as a supplement for astronauts [63]. Many studies have reported that vitamin D has a positive effect on astronauts, our results support previous studies. In addition, bioinformatics analysis showed that EGCG may have the potential to reduce susceptible viral disease infection (HSV, EBV, KSHV, HIV and HBV) risk in space environments. HSV, KSHV and EBV are spore rash viruses, HIV and HBV are retroviruses. EGCG has different modes of antiviral action and has been demonstrated in a variety of viridae families including the above-mentioned viruses [64,65,66,67,68].

In conclusion, the results of this study can provide a reference for formulating dietary and medical programs for astronauts during space missions. However, due to the small sample size of spatial data, the influence brought by individual differences cannot be excluded from the study.

## Figures and Tables

**Table 1:** Nutrients that significantly interact with multiple viral infections.

Virus	Epigallocatechin-3-Gallate	Vitamin D	Ethyl Alcohol	Lycopene
HSV Infection	1.79 × 10^−10^	1.55 × 10^−4^	7.11 × 10^−5^	
EBV Infection	5.40 × 10^−17^	3.95 × 10^−3^	1.91 × 10^−15^	
KSHV Infection	2.94 × 10^−17^	1.22 × 10^−4^	9.49 × 10^−9^	1.52 × 10^−3^
HPV Infection	1.07 × 10^−2^		5.19 × 10^−4^	
HIV Infection	1.74 × 10^−14^	1.05 × 10^−7^	1.25 × 10^−5^	1.43 × 10^−2^
HBV Infection	7.86 × 10^−17^	6.18 × 10^−5^	3.76 × 10^−15^	9.26 × 10^−3^

Nutrients that significantly interact with multiple viral infections and adjust *p*-values (calculated by hypergeometric distribution test) less than 0.05 are shown in the table. Adjust *p*-value was obtained by hypergeometric distribution test of the gene set related to nutrients and the gene set related to viral infection. The lower the adjusted *p*-value, the more probable the nutrient interacts with the genes involved in the viral infection pathway.

**Table 2:** 2SMR results for nutrients and indicator levels of viral infections.

Nutrients	Trait	HPV E7 Type 18 (*p*-Value)	EBNA IgG Levels (*p*-Value)	HBs IgG Levels (*p*-Value)
Vitamin D	Vitamin D levels	0.3050	0.0283	0.0069
Calcium	Calcium levels	0.0039	0.3088	0.8635
Selenium	Blood trace element (Se levels)	0.4299	0.0715	0.2474

*p*-value represents the correlation between nutrient and indicator levels of viral infection. *p*-value less than 0.05 indicated a significant correlation between nutrient and indicator levels of viral infection but did not reflect a causal relationship.

## Data Availability

Not applicable.
